# Role of Circulating Lipids in Mediating the Diabetogenic Effect of Obesity

**DOI:** 10.3390/biomedicines14010011

**Published:** 2025-12-20

**Authors:** Yutang Wang, Yan Fang, Fadi J. Charchar, Grant R. Drummond, Christopher G. Sobey

**Affiliations:** 1 Health Innovation and Transformation Centre, Federation University Australia, Ballarat, VIC 3350, Australia; 2 Centre for Cardiovascular Biology and Disease Research, La Trobe Institute for Molecular Science, La Trobe University, Bundoora, VIC 3086, Australia; 3 Department of Microbiology, Anatomy, Physiology & Pharmacology, School of Agriculture, Biomedicine & Environment, La Trobe University, Bundoora, VIC 3086, Australia

**Keywords:** lipids, type 2 diabetes, triglyceride, metabolic disease

## Abstract

**Background/Objectives**: Obesity is a major risk factor for diabetes, but the underlying mechanisms remain incompletely understood. Obesity is associated with alterations in circulating lipids. This study aimed to determine whether, and to what extent, circulating lipids mediate the diabetogenic effect of obesity. **Methods**: This mediation analysis included 26,627 adult participants. Parallel mediation analysis included total cholesterol, high-density lipoprotein (HDL) cholesterol, and triglycerides as simultaneous mediators. Low-density lipoprotein (LDL) cholesterol was excluded from the parallel model due to collinearity with total cholesterol and was assessed separately using simple mediation analysis adjusted for confounders. **Results**: After adjustment for tested confounders, parallel mediation analysis showed that increases in triglycerides and reductions in HDL cholesterol mediated 24.0% (indirect effect coefficient = 0.23; 95% CI: 0.20–0.26; *p* < 0.05) and 3.8% (indirect effect coefficient = 0.04; 95% CI: 0.01–0.06; *p* < 0.05) of the diabetogenic effect of obesity, respectively. An increase in total cholesterol modestly attenuated the diabetogenic effect of obesity by 2.3% (indirect effect coefficient = −0.02; 95% CI: −0.03 to −0.01; *p* < 0.05), a magnitude that is unlikely to be clinically meaningful. Simple mediation analysis indicated that LDL cholesterol was not a significant mediator. **Conclusions**: Triglycerides are the most influential circulating lipid in mediating the diabetogenic effect of obesity, accounting for 24% of the total effect. Targeting triglyceride levels might represent an underrecognized therapeutic strategy to reduce obesity-related diabetes risk.

## 1. Introduction

Obesity is defined as excessive fat accumulation in the body that can impair health [1]. Over the past three decades (1990–2021), global adult obesity prevalence has risen dramatically—by 105% in females (from 10.2% to 20.8%) and by 155% in males (from 5.8% to 14.8%) [2]. The World Health Organization (WHO) has classified obesity as a global epidemic [3]. According to the WHO, 890 million adults worldwide are living with diabetes, representing 16% of the global population [4]. Obesity in adults is particularly common in high-income countries, with prevalence rates of 28% in the UK [5], 29.5% in Canada [6], 32% in Australia [7], and 41.9% in the United States [8]. It is projected that by 2050, the number of adults with obesity will reach 1.95 billion [2].

Obesity has wide-ranging consequences for health and well-being [1]. Individuals with obesity often experience stigma and discrimination, which negatively impact quality of life [9,10] and increase the risk of depression [11]. Excess adipose tissue can also impair organ and tissue function; for example, it can damage joints, leading to osteoarthritis, pain, and reduced mobility [12]. Furthermore, obesity is a major risk factor for numerous diseases, including liver disease [13], chronic kidney disease, cardiovascular disease, and cancer [14,15].

Of particular concern, obesity substantially increases the risk of diabetes [16]. Currently, diabetes affects 537 million people worldwide [17], and both its prevalence and incidence continue to rise [17,18]. Diabetes can lead to severe complications, including blindness, kidney failure, heart attacks, stroke, and lower-limb amputation [19]. The World Obesity Federation and the International Diabetes Federation estimate that obesity accounts for 43% of type 2 diabetes cases [20], which itself represents approximately 90% of all diabetes diagnoses [21]. Both organizations emphasize that halting the global rise in type 2 diabetes requires prioritizing action on obesity [20].

Obesity contributes to diabetes through multiple mechanisms [16,22,23]. A key pathway involves obesity-induced insulin resistance and β-cell dysfunction [16], driven by increased oxidative stress and chronic inflammation [22,23]. Obesity is also strongly associated with dyslipidemia, characterized by elevated triglycerides [24], increased total cholesterol [25], higher low-density lipoprotein (LDL) cholesterol [26], and reduced high-density lipoprotein (HDL) cholesterol [27,28,29]. Most individuals with obesity (60–70%) exhibit dyslipidemia [30]. However, the extent to which these circulating lipids mediate the diabetogenic effect of obesity remains unclear.

To address this question, we analyzed data from 26,627 US adults who participated in the National Health and Nutrition Examination Survey (NHANES) between 1988 and 2014. Circulating lipids assessed included total cholesterol, HDL cholesterol, LDL cholesterol, and triglycerides.

## 2. Materials and Methods

### 2.1. Study Participants

**Figure 1:**
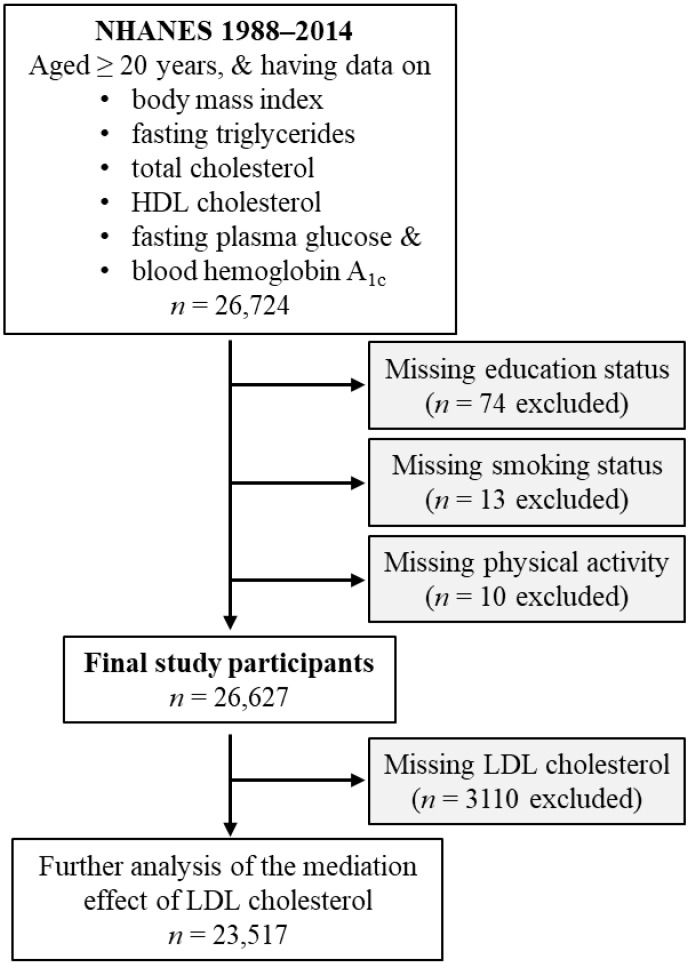
Flow diagram of the study participants. HDL, high-density lipoprotein; LDL, low-density lipoprotein; NHANES, National Health and Nutrition Examination Survey.

This study included US civilian noninstitutionalized individuals who attended the NHANES from 1988 to 2014. These surveys were organized by the Centers for Disease Control and Prevention (CDC) [31]. The inclusion criteria of the current study were age of 20 years or older, and the availability of the following data: body mass index, fasting triglycerides, total cholesterol, HDL cholesterol, fasting plasma glucose, and blood hemoglobin A_1c_ (HbA_1c_). A total of 26,724 participants met these criteria. The following individuals with unknown education status (*n* = 74), unknown smoking status (*n* = 13), or unknown physical activity status (*n* = 10), were excluded. Therefore, 26,627 participants were included in the final analysis (Figure 1).

In a further analysis investigating the effect of LDL cholesterol in mediating the diabetogenic effect of obesity, 3110 participants were excluded due to missing LDL cholesterol values. Therefore, a total of 23,517 participants were included in this further analysis (Figure 1).

### 2.2. Exposure Variable

The exposure variable was obesity, which was defined as a body mass index of 30 kg/m^2^ or above [32,33]. In addition, body mass index (continuous) was used as the exposure variable in further analysis.

### 2.3. Outcome Variable

Diabetes was the outcome variable. It was classified by one of the following conditions: an HbA_1c_ value of ≥6.5%, a fasting plasma glucose value of ≥126 mg/dL, a 2-h plasma glucose value during oral glucose tolerance test of ≥200 mg/dL, the use of anti-diabetic drugs, or self-reported diabetes diagnosis [34,35].

### 2.4. Candidate Mediators

The mediators assessed in this study included total cholesterol, HDL cholesterol, LDL cholesterol, and triglycerides. Fasting blood samples were collected from participants who had fasted for at least 8 h after their last caloric intake [35,36,37].

Total cholesterol was measured enzymatically using a series of coupled reactions: cholesteryl esters were hydrolyzed to cholesterol by cholesterol esterase; cholesterol was then oxidized by cholesterol oxidase, producing hydrogen peroxide; and hydrogen peroxide was converted into a red dye by peroxidase in the presence of 4-aminophenazone and phenol. The color intensity, directly proportional to cholesterol concentration, was determined photometrically at 500 nm [38].

HDL cholesterol was measured directly without removing apoB-containing lipoproteins [39]. A blocking reagent rendered LDL, very low-density lipoprotein (VLDL), and chylomicrons non-reactive with the enzymatic cholesterol reagent under assay conditions, effectively excluding them from detection. HDL cholesterol esters were converted to cholesterol by polyethylene glycol (PEG)-modified cholesterol esterase, then oxidized by cholesterol oxidase to Δ4-cholestenone and hydrogen peroxide. In the presence of peroxidase, hydrogen peroxide reacted with 4-amino-antipyrine and N-(2-hydroxy-3-sulfopropyl)-3,5-dimethoxyaniline (HSDA) to form a purple-blue dye. The color intensity, proportional to HDL cholesterol concentration, was measured photometrically.

Triglycerides were measured enzymatically according to the published method [40]. LDL cholesterol was not directly measured [41]; instead, it was calculated using the Friedewald formula [42].

### 2.5. Confounding Variables

The study included a broad range of confounders, including age (continuous), sex, ethnicity, education status, income status, survey periods, physical activity, alcohol consumption status, smoking status, hypertension, and family history of diabetes [43,44,45].

### 2.6. Statistical Analyses

Baseline characteristics of participants were summarized as follows: categorical variables were presented as numbers (percentages), non-normally distributed continuous variables as medians (interquartile ranges), and normally distributed continuous variables as means (standard deviations) [46]. Differences in categorical variables were assessed using Pearson’s chi-square test [47], while differences in continuous variables were evaluated using Student’s *t*-test for normally distributed variables and the Mann–Whitney U test for non-normally distributed variables [48]. Correlations among the lipids were analysed using bivariate Pearson correlation analysis.

**Figure 2:**
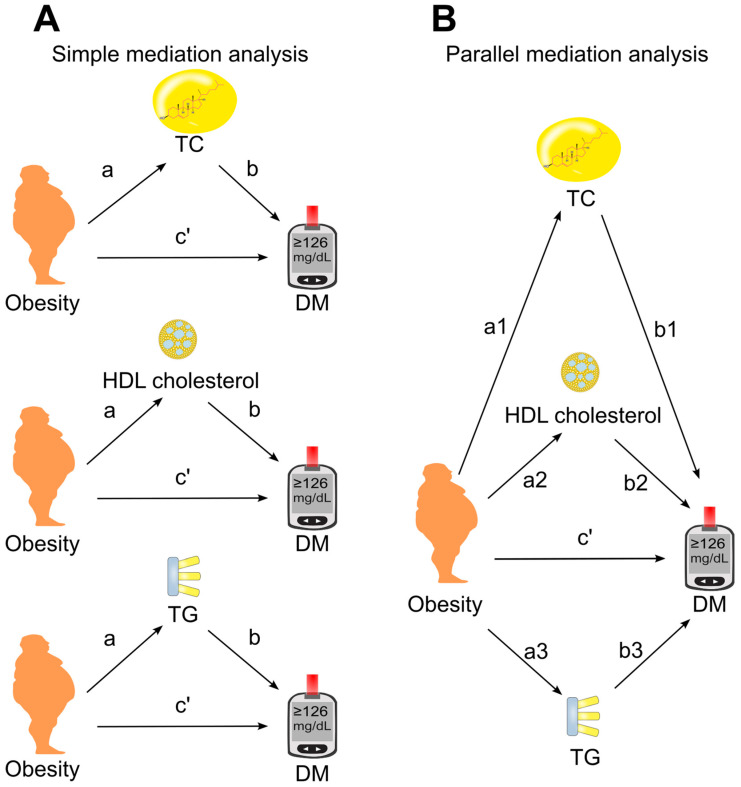
Models of mediation. (**A**), Simple mediation model. Total cholesterol, HDL cholesterol, or triglycerides was added as single mediator between obesity and diabetes. (**B**), Parallel mediation model. Total cholesterol, HDL cholesterol, and triglycerides were added simultaneously to the same model. a, association coefficient of obesity with mediator; b, association coefficient of mediator with diabetes; c′, direct effect which measures the association coefficient of obesity with diabetes in the presence of mediator(s). DM, diabetes; HDL, high-density lipoprotein; TC, total cholesterol; TG, triglycerides.

The association of obesity with diabetes was examined using binary logistic regression [49]. Mediation analysis was performed using the PROCESS Version 4.3 Macro for SPSS [50,51] with Model number 4. The 95% confidence interval (CI) for the indirect effect was estimated via bootstrapping using 5000 samples without centering for the construction of products [52,53]. First, a simple mediation analysis was conducted (Figure 2A), where candidate mediators (total cholesterol, HDL cholesterol, and triglycerides) were analyzed individually to estimate their separate mediation effects on the obesity–diabetes association. Subsequently, a parallel mediation analysis was performed (Figure 2B), in which all three mediators were included simultaneously in the same model.

Further analyses assessed the mediation effect of LDL cholesterol. Participants lacking LDL cholesterol data (*n* = 3310) were excluded, leaving 23,517 participants for this analysis (Figure 1). Because LDL cholesterol was highly correlated with total cholesterol (Pearson correlation coefficient = 0.917, Table 1), it was excluded from the parallel mediation model to avoid collinearity. Instead, its mediation effect was examined using simple mediation analysis, adjusting for all tested confounders as well as HDL cholesterol and triglycerides.

Association coefficients were derived from mediation analysis (Figure 2). Coefficient a represented the association of obesity with the tested mediator, while coefficient b reflected the association of the mediator with diabetes. The direct effect (c′) was the association of obesity with diabetes after accounting for the mediator(s). The indirect effect, also referred to as the mediation effect, was calculated as a × b [34].

A mediation effect was considered statistically significant (*p* < 0.05) if the 95% CI did not include zero [54]. The following formula was used to calculate proportion mediated: Proportion mediated = a × b/(a × b + c′). This metric indicates the extent to which the tested mediator explains the effect of obesity on diabetes [34,55].

Additional analyses were performed by replacing obesity with body mass index (continuous) in the mediation models. Sensitivity analyses were conducted by excluding participants who were using anti-diabetic or lipid-lowering medications or by omitting individuals who participated in surveys prior to 1999. NHANES sampling weights were not applied in any of the analyses.

Triglycerides, body mass index, total cholesterol, and HDL cholesterol were natural log-transformed to improve data distribution prior to inclusion in regression and mediation models [56]. Using obesity as a categorical variable enhances interpretability, making the findings more accessible to researchers, clinicians, and the general public. Therefore, the primary analysis in this study was conducted using obesity as a categorical variable. Two-tailed *p*-values < 0.05 were regarded as statistical significance. SPSS (version 27.0, IBM SPSS Statistics for Windows, Armonk, NY, USA, IBM Corporation) was used to perform all the statistical analyses.

## 3. Results

### 3.1. General Characteristics

This study included 26,627 adults, with a mean age of 48 years. Among these participants, 3958 individuals (14.9%) had diabetes, and 8425 individuals (31.6%) were obese. Compared to non-obese individuals, those with obesity had a higher likelihood of diabetes and hypertension, higher levels of triglycerides, higher levels of total cholesterol, higher levels of LDL cholesterol, lower levels of HDL cholesterol, and less physical activity (Table 2).

### 3.2. Association of Obesity with Diabetes Diagnosis

Obesity was associated with a 2.44-fold higher odds ratio for diabetes (Model 4, Table 3) after adjustment for risk factors except total cholesterol, HDL cholesterol, and triglycerides (i.e., three tested mediators). After further adjustment for these tested mediators, obesity remained associated with a higher risk of diabetes (Model 8, Table 3), suggesting that any mediation by total cholesterol, HDL cholesterol, or triglycerides is partial rather than complete [57].

### 3.3. Role of Circulating Lipids in Mediating the Effect of Obesity on Diabetes

**Figure 3:**
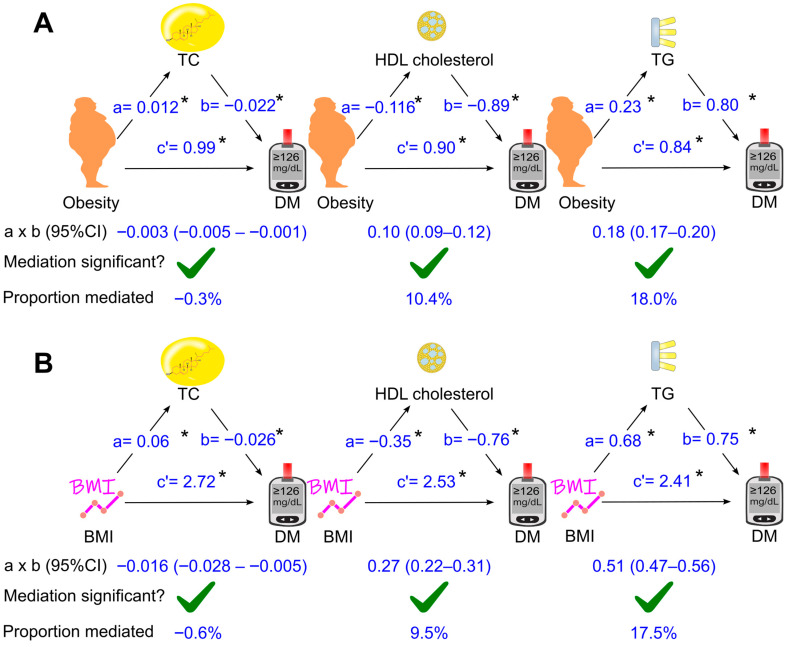
Simple mediation analysis. Total cholesterol, HDL cholesterol, or triglycerides was added separately into the model using obesity (**A**) or BMI (**B**) as the exposure variable. a, association coefficient of obesity with mediator or that of BMI with mediator; b, association coefficient of mediator with diabetes; c′, association coefficient of obesity with diabetes or that of BMI with diabetes in the presence of mediator. BMI, body mass index; CI, confidence interval; DM, diabetes; HDL, high-density lipoprotein; TC, total cholesterol; TG, triglycerides. Green ticks indicate statistical significance. *, *p* <0.05.

**Figure 4:**
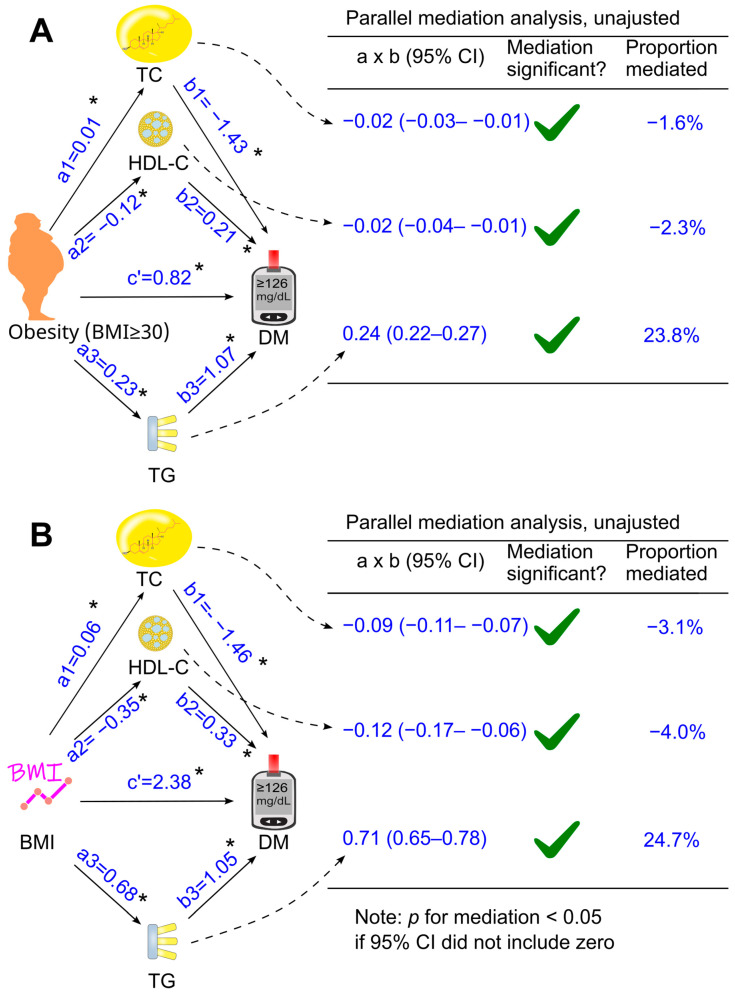
Parallel mediation analysis without adjustment. Total cholesterol, HDL cholesterol, and triglycerides were added simultaneously into the same model using obesity (**A**) or BMI (**B**) as the exposure variable. These analyses were not adjusted for confounders. a, association coefficient of obesity with the tested mediator or that of BMI with the tested mediator; b, association coefficient of the tested mediator with diabetes; c′, association coefficient of obesity with diabetes or that of BMI with diabetes. BMI, body mass index; CI, confidence interval; DM, diabetes; HDL-C, high-density lipoprotein cholesterol; TC, total cholesterol; TG, triglycerides. Green ticks indicate statistical significance. *, *p* <0.05.

**Figure 5:**
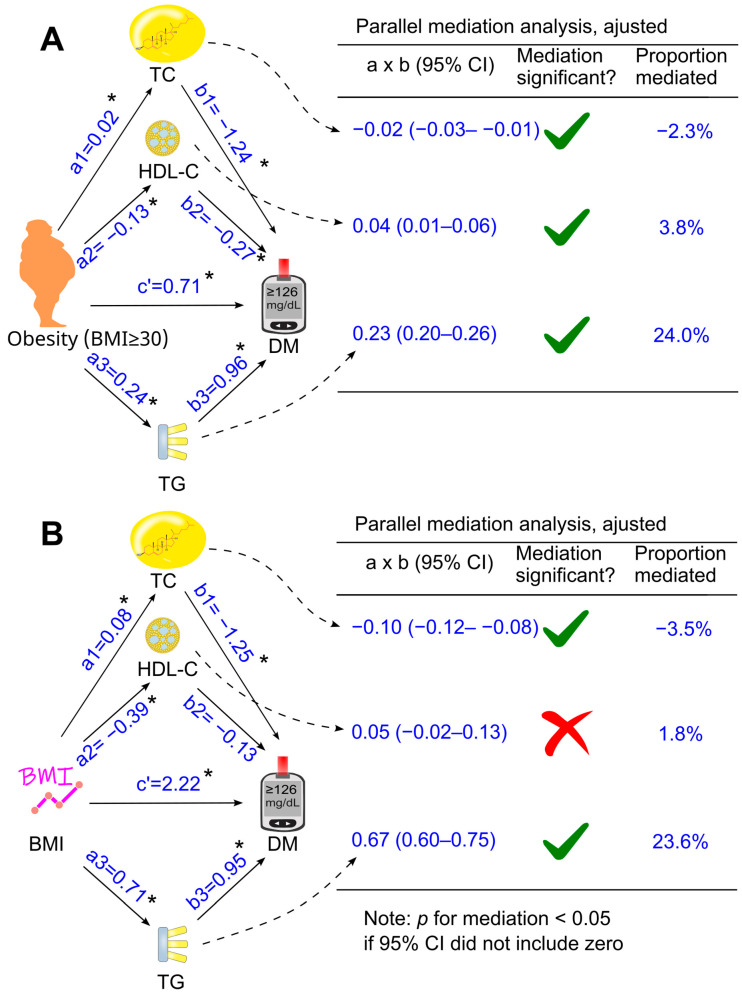
Adjusted parallel mediation analysis. Total cholesterol, HDL cholesterol, and triglycerides were placed simultaneously into the same model using obesity (**A**) or BMI (**B**) as the exposure variable. This analysis was adjusted for confounding factors, including age, sex, ethnicity, poverty-income ratio, education status, survey period, lifestyle confounding factors (physical activity, alcohol consumption status, and smoking status), clinical confounding factors (hypertension and family history of diabetes). Abbreviations: a, association coefficient of obesity with mediator, or that of BMI with mediator; b, association coefficient of mediator with diabetes; c′, association coefficient of obesity with diabetes or that of BMI with diabetes in the presence of mediators. BMI, body mass index; CI, confidence interval; DM, diabetes; HDL-C, high-density lipoprotein cholesterol; TC, total cholesterol; TG, triglycerides. Green ticks indicate statistical significance, while red crosses indicate non-significance. *, *p* <0.05.

The mediation coefficients of total cholesterol, HDL cholesterol, and triglycerides for the effect of obesity on diabetes are displayed in Figure 3, Figure 4 and Figure 5. When total cholesterol, HDL cholesterol, and triglycerides was added separately into the analysis model, they all mediated the association of obesity with diabetes (Figure 3A), with triglycerides as the most dominant mediator, accounting for 18% of the diabetogenic effect of obesity (Figure 3A).

When total cholesterol, HDL cholesterol, and triglycerides were included together as mediators in a parallel mediation model, all three parameters continued to mediate the relationship between obesity and diabetes, both without adjustment (Figure 4A) and after adjusting for confounders (Figure 5A). Increases in triglycerides and reductions in HDL cholesterol mediated 24.0% and 3.8% of the total effect after adjustment, respectively, whereas an increase in total cholesterol modestly attenuated the diabetogenic effect of obesity by 2.3%, a magnitude unlikely to be clinically meaningful (Figure 5A).

Similar results were observed after excluding participants taking anti-diabetic or lipid-lowering medications (Supplementary Figure S1). Triglycerides remained the most influential circulating lipid, mediating 21% of the diabetogenic effect of obesity. A reduction in HDL cholesterol explained 8% of the effect, whereas the small effect of total cholesterol was no longer statistically significant (Supplementary Figure S1).

Similar results were observed after excluding individuals who participated in surveys prior to 1999 (Supplementary Figure S2). Triglycerides remained the most influential circulating lipid, mediating 24.5% of the diabetogenic effect of obesity. In contrast, the reduction in HDL cholesterol did not significantly contribute to this mediation effect, and the attenuating influence of total cholesterol remained minimal (Supplementary Figure S2).

### 3.4. Role of Circulating Lipids in Mediating the Effect of Body Mass Index on Diabetes

Further analyses were conducted when obesity was replaced with a continuous variable, i.e., body mass index (Figure 3B, Figure 4B and Figure 5B). After adjustment for all the tested confounders, the parallel mediation analysis showed that triglycerides remained the most dominant mediator among the three tested mediators, mediated 23.6% of the association between body mass index and diabetes, and total cholesterol slightly attenuated the association by 3.5% (Figure 5B). However, HDL cholesterol did not mediate the effect of body mass index on diabetes (Figure 5B, *p* > 0.05)

### 3.5. Further Analyses of the Role of LDL Cholesterol in Mediating the Effect of Obesity (Or Body Mass Index) on Diabetes

**Figure 6:**
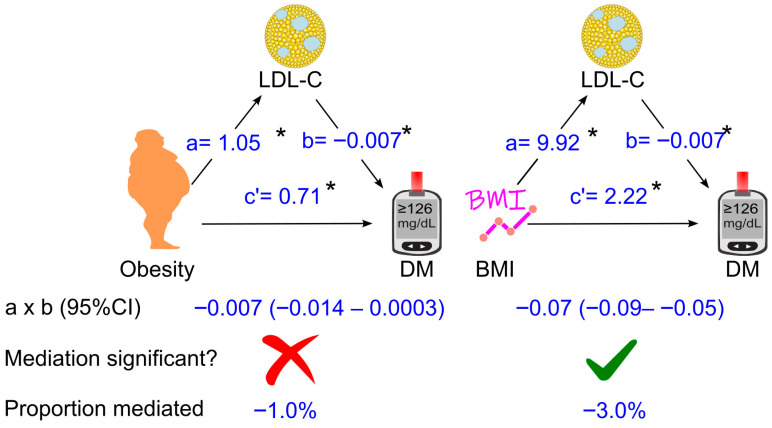
Association coefficients of LDL cholesterol for mediating the effect of obesity or BMI on diabetes in 23,517 participants. The analysis was adjusted for age, sex, ethnicity, poverty-income ratio, education status, survey period, lifestyle confounding factors (physical activity, alcohol consumption status, and smoking status), clinical confounding factors (hypertension and family history of diabetes), high-density lipoprotein (HDL) cholesterol, and triglycerides. Abbreviations: a, association coefficient of obesity with LDL cholesterol, or that of BMI with LDL cholesterol; b, association coefficient of LDL cholesterol with diabetes; c′, association coefficient of obesity with diabetes or that of BMI with diabetes in the presence of mediators and confounders. BMI, body mass index; CI, confidence interval; DM, diabetes. Green ticks indicate statistical significance, while red crosses indicate non-significance. *, *p* <0.05.

Further analyses were conducted in a sub-cohort of 23,517 participants after 3110 participants were excluded due to missing LDL cholesterol. After adjustment for all the tested confounders, an increase in LDL cholesterol slightly attenuated the diabetogenic effect of obesity by 1%, which was not significant (*p* > 0.05, Figure 6). However, when obesity was replaced by the body mass index, an increase in LDL cholesterol attenuated the diabetogenic effect of higher BMI by 3% (*p* < 0.05, Figure 6). Similar findings were observed after excluding participants who were taking anti-diabetic or lipid-lowering medications (Supplementary Figure S3) and after omitting individuals who participated in surveys prior to 1999 (Supplementary Figure S4). Notably, LDL cholesterol did not substantially contribute to the diabetogenic effect of obesity, with proportions mediated of −0.4% and −1.1%, respectively (Supplementary Figures S3 and S4).

## 4. Discussion

Utilizing a large sample of US adults (*n* = 26,627), this study found that the increase in triglycerides and the decrease in HDL cholesterol might mediate 24.0% (*p* < 0.05) and 3.8% (*p* < 0.05) of the diabetogenic effect of obesity, respectively, after adjusting for tested confounders. In contrast, an increase in total cholesterol modestly attenuated the diabetogenic effect of obesity by 2.3% (*p* < 0.05), a magnitude that is unlikely to be clinically meaningful. In addition, LDL cholesterol was not a significant factor mediating the diabetogenic effect of obesity. These findings indicate that triglycerides are the most influential circulating lipid mediating the diabetogenic effect of obesity.

Our analysis indicated that an increase in total cholesterol attenuated the diabetogenic effect of obesity by 2.3%. Although statistically significant, this effect is unlikely to be clinically meaningful. LDL cholesterol did not influence the diabetogenic effect of obesity. However, when BMI was used instead of obesity, an increase in LDL cholesterol moderately attenuated the diabetogenic effect of higher BMI by 3.0%. Despite statistical significance, this association also appears to have negligible clinical relevance.

Overall, these results suggest that although obesity is associated with increased total and LDL cholesterol, these lipids do not play a major role in mediating its diabetogenic effect. They may even provide slight protection against obesity-related diabetes risk. The mechanisms underlying these observations remain unclear. Nevertheless, our findings align with existing literature showing that lowering cholesterol and LDL cholesterol with statins slightly increases type 2 diabetes risk by approximately 10–12% [58,59,60,61,62]. Statins reversibly and competitively inhibit HMG-CoA reductase, the rate-limiting enzyme in cholesterol biosynthesis [63], thereby reducing cholesterol production and circulating total cholesterol. Additionally, statin-induced reductions in intracellular cholesterol upregulate LDL receptors (LDLR) in the liver and peripheral tissues, enhancing LDL clearance and lowering circulating LDL cholesterol [62,64].

This study also found that a reduction in HDL cholesterol mediated 3.8% of the diabetogenic effect of obesity. Obesity was associated with decreased HDL cholesterol, consistent with previous reports [27,28,29]. This reduction contributed to increased diabetes risk, supporting evidence that HDL cholesterol protects against type 2 diabetes [65,66] through mechanisms such as stimulating pancreatic insulin synthesis and secretion [67] and enhancing skeletal muscle glucose uptake [68]. Similarly, treatment with cholesteryl ester transfer protein (CETP) inhibitors to raise HDL cholesterol improved glycemic control in patients with type 2 diabetes [69].

The reduction in HDL cholesterol appeared to play only a minor role (3.8%) in mediating the diabetogenic effect of obesity, and this effect disappeared when BMI replaced obesity in the analysis. Notably, the use of anti-diabetic and lipid-lowering medications may influence this relationship. In our sensitivity analysis excluding individuals on these drugs, a decrease in HDL cholesterol mediated 8% of the diabetogenic effect of obesity and 5.5% of that associated with higher BMI.

Triglycerides, unlike total cholesterol, LDL cholesterol, and HDL cholesterol, played a substantially greater role in mediating the diabetogenic effect of obesity, accounting for 24% of the total effect. We observed that obesity was associated with elevated triglyceride levels, consistent with previous reports [24,26,70]. Furthermore, this increase in triglycerides was linked to a higher risk of diabetes, in agreement with existing literature [36,71,72]. Mechanistically, elevated circulating triglycerides promote intracellular triglyceride accumulation and reduce the capacity of cells to store excess glucose as triglycerides, thereby inducing insulin resistance [73]. In addition, high triglyceride levels contribute to β-cell dysfunction [74,75] and apoptosis [76], as well as increased hepatic gluconeogenesis [73,77,78]. Collectively, these mechanisms underscore the role of triglycerides in diabetes development.

The significance of triglycerides in mediating the diabetogenic effect of obesity is further supported by evidence from bariatric surgery. Weight loss induced by bariatric surgery improves glycemic control and often leads to diabetes remission [79,80,81]. Notably, these improvements occur without significant changes in total cholesterol [79,81] or LDL cholesterol [80,81], reinforcing our observation that these lipids play only a minor role in mediating obesity’s diabetogenic effect. Although bariatric surgery is frequently associated with increased HDL cholesterol [79,80], HDL cholesterol does not appear to be a major contributor to the anti-diabetic effect. For example, Genua et al. reported that blood glucose levels declined within three months post-surgery, while HDL cholesterol decreased during this period; although HDL cholesterol subsequently increased and remained elevated for up to five years, changes in body mass index were not associated with changes in HDL cholesterol [81].

In contrast, bariatric surgery consistently reduces circulating triglyceride levels [79,80,82,83]. This reduction parallels a decrease in intracellular triglyceride deposition in the liver and skeletal muscle [84]. These findings suggest that triglycerides may play a central role in mediating the anti-diabetic effects of weight loss, consistent with our observation that triglycerides accounted for 24% of the total effect of obesity.

Evidence from dietary energy restriction-induced weight loss further supports our findings. Lim et al. [85] reported that dietary energy restriction led to weight loss and reduced blood glucose levels within one week of intervention. However, at this time point, no changes in HDL cholesterol or LDL cholesterol were observed, suggesting that these lipids may not play a major role in mediating the antidiabetic effect of dietary energy restriction-induced weight loss—consistent with our results. In contrast, Lim et al. [85] found that the antidiabetic effect of dietary energy restriction-induced weight loss was accompanied by a reduction in circulating triglycerides. Moreover, a decline in intracellular triglyceride content in the pancreas was associated with restored insulin secretion, while a similar decline in the liver corresponded with reduced hepatic glucose production and lower fasting plasma glucose [85,86]. These findings suggest that lowering circulating triglycerides may contribute significantly to the antidiabetic effect of dietary energy restriction-induced weight loss.

Taken together, our results and evidence from weight-loss studies [79,80,81,82,83,84,85,86], support the notion that circulating triglycerides—rather than total cholesterol, HDL cholesterol, or LDL cholesterol—play a key role in mediating the diabetogenic effect of obesity. Therefore, targeting circulating triglycerides may represent a promising therapeutic strategy to reduce obesity-induced diabetes risk. Research indicates that lowering triglycerides with fenofibrate improves insulin sensitivity and reduces plasma glucose in mice [87]. Furthermore, fenofibrate has been shown to protect against T2DM in mice [88] and to slow the progression of albuminuria [89] and retinopathy [90] in patients with T2DM. In addition, bezafibrate has been reported to reduce the incidence of T2DM in humans [91].

Strengths of this study include its large sample size and adjustment for multiple confounding factors. However, the findings were based on US participants and may not be generalizable to other populations. In addition, although this study adjusted for a broad range of confounding factors, the presence of unmeasured confounders cannot be ruled out and may have influenced the results. Moreover, because diabetes can influence lipid levels and BMI, the possibility of reciprocal mediation cannot be excluded. A longitudinal study is warranted to confirm these findings.

## 5. Conclusions

This study demonstrates that among circulating lipids, triglycerides play the central mediating role in the diabetogenic effect of obesity. Consequently, targeting circulating triglycerides might be an underrecognized therapeutic approach for managing obesity-related diabetes.

## Figures and Tables

**Table 1:** Bivariate Pearson correlation coefficient among the circulating lipids.

	Total Cholesterol	HDL Cholesterol	LDL Cholesterol
HDL cholesterol	0.154		
LDL cholesterol	0.917	−0.070	
Triglycerides	0.367	−0.317	0.178

HDL, high-density lipoprotein; LDL, low-density lipoprotein.

**Table 2:** Characteristics of the 26,627 participants, stratified according to obesity ^1^.

	Non Obese	Obese	Overall	*p* Value
Sample size	18,202	8425	26,627	NA
BMI, kg/m^2^, median (IQR)	25 (23–27)	34 (32–38)	27 (24–31)	<0.001
Diabetes, *n* (%)	1926 (10.6)	2032 (24.1)	3958 (14.9)	<0.001
Glucose, mg/dL, median (IQR)	95 (89–104)	101 (94–114)	97 (90–106)	<0.001
HbA_1c_, %, median (IQR)	5.3 (5.1–5.6)	5.6 (5.3–6.0)	5.4 (5.1–5.8)	<0.001
TC, mg/dL, median (IQR)	196 (169–225)	198 (172–227)	196 (170–225)	<0.001
HDL cholesterol, mg/dL, median (IQR)	52 (43–64)	46 (39–56)	50 (42–61)	<0.001
LDL cholesterol ^2^, mg/dL, mean (SD)	119.3 (36.8)	121.3 (36.6)	120.0 (36.8)	<0.001
Triglycerides, mg/dL, median (IQR)	102 (72–150)	131 (91–189)	110 (77–163)	<0.001
Age, y, mean (SD)	48 (19)	49 (17)	48 (19)	<0.001
Sex (male), *n* (%)	9248 (50.8)	3503 (41.6)	12,751 (47.9)	<0.001
Ethnicity, *n* (%)				
Non-Hispanic white	8522 (46.8)	3464 (41.1)	11,986 (45)	<0.001
Non-Hispanic black	3567 (19.6)	2265 (26.9)	5832 (21.9)	
Hispanic	4963 (27.3)	2463 (29.2)	7426 (27.9)	
Other	1150 (6.3)	233 (2.8)	1383 (5.2)	
Education status, *n* (%)				
<High School	5790 (31.8)	2786 (33.1)	8576 (32.2)	0.01
High School	4651 (25.6)	2209 (26.2)	6860 (25.8)	
>High School	7761 (42.6)	3430 (40.7)	11,191 (42.0)	
Poverty-income ratio, *n* (%)				
<130%	5008 (27.5)	2585 (30.7)	7593 (28.5)	<0.001
130–349%	6714 (36.9)	3136 (37.2)	9850 (37.0)	
≥350%	4929 (27.1)	2032 (24.1)	6961 (26.1)	
Unknown	1551 (8.5)	672 (8.0)	2223 (8.3)	
Physical activity, *n* (%)				
Active	5351 (29.4)	1696 (20.1)	7047 (26.5)	<0.001
Insufficiently active	6807 (37.4)	3115 (37.0)	9922 (37.3)	
Inactive	6044 (33.2)	3614 (42.9)	9658 (36.3)	
Alcohol consumption, *n* (%)				
0 drink/week	2957 (16.2)	1775 (21.1)	4732 (17.8)	<0.001
<1 drink/week	3846 (21.1)	2166 (25.7)	6012 (22.6)	
1–6 drinks/week	4004 (22.0)	1392 (16.5)	5396 (20.3)	
≥7 drinks/week	2606 (14.3)	829 (9.8)	3435 (12.9)	
Unknown	4789 (26.3)	2263 (26.9)	7052 (26.5)	
Smoking status, *n* (%)				
Past smoker	4435 (24.4)	1587 (18.8)	6022 (22.6)	<0.001
Current smoker	4413 (24.2)	2260 (26.8)	6673 (25.1)	
Nonsmoker	9354 (51.4)	4578 (54.3)	13,932 (52.3)	
Hypertension, *n* (%)				
No	11,963 (65.7)	3919 (46.5)	15,882 (59.6)	<0.001
Yes	5991 (32.9)	4377 (52.0)	10,368 (38.9)	
Unknown	248 (1.4)	129 (1.5)	377 (1.4)	
Family history of diabetes, *n* (%)				
Yes	7239 (39.8)	4340 (51.5)	11,579 (43.5)	<0.001
No	10,626 (58.4)	3931 (46.7)	14,557 (54.7)	
Unknown	337 (1.9)	154 (1.8)	491 (1.8)	

^1^ Obesity was classified as a BMI value of 30 kg/m^2^ or above. ^2^ LDL cholesterol data were generated from 23,517 participants, as 3110 participants did not have the data. Abbreviations: BMI, body mass index; HbA_1c_, hemoglobin A_1c_; HDL, high-density lipoprotein; IQR, interquartile range; LDL, low-density lipoprotein; *n*, number; NA, not applicable; SD, standard deviation; TC, total cholesterol.

**Table 3:** Obesity-associated odds ratio for diabetes in 26,627 individuals.

Models	Odds Ratio	95% CI	*p* Value
Model 1	2.69	2.51–2.88	<0.001
Model 2	3.11	2.88–3.35	<0.001
Model 3	2.84	2.63–3.07	<0.001
Model 4	2.44	2.25–2.65	<0.001
Model 5 (Model 4 + TC)	2.44	2.25–2.65	<0.001
Model 6 (Model 4 + HDL cholesterol)	2.14	1.97–2.32	<0.001
Model 7 (Model 4 + TG)	2.13	1.96–2.31	<0.001
Model 8 (Model 4 + TC + HDL cholesterol + TG)	2.03	1.87–2.21	<0.001

CI, confidence interval; HDL, high-density lipoprotein; TC, total cholesterol; TG, triglycerides. Model 1: Not adjusted; Model 2: Adjusted for age, sex, and ethnicity; Model 3: Adjusted for factors in Model 2 plus poverty-income ratio, education status, physical activity, alcohol consumption status, smoking status, and survey period; Model 4: Adjusted for factors in Model 3 plus hypertension and family history of diabetes.

## Data Availability

All data included in this study are publicly available on the NHANES website (https://www.cdc.gov/nchs/nhanes/index.htm; accessed on 1 July 2025).

## Supplementary Materials

The following supporting information can be downloaded at: https://www.mdpi.com/article/10.3390/biomedicines14010011/s1, Supplementary Figure S1: Sensitivity analysis of the parallel analysis after excluding participants who were taking anti-diabetic or lipid-lowering drugs; Supplementary Figure S2: Sensitivity analysis of the parallel analysis after excluding participants who participated in surveys prior to 1999; Supplementary Figure S3: Sensitivity analysis of association coefficients of LDL cholesterol for mediating the effect of obesity or BMI on diabetes after excluding those who were taking anti-diabetic or lipid-lowering drugs; Supplementary Figure S4: Sensitivity analysis of association coefficients of LDL cholesterol for mediating the effect of obesity or BMI on diabetes after excluding those who participated in surveys prior to 1999.

## Abbreviations

The following abbreviations are used in this manuscript:

BMI	Body mass index
CDC	Centers for Disease Control and Prevention
CETP	Cholesteryl ester transfer protein
CI	Confidence interval
DM	Diabetes
HbA1c	Hemoglobin A1c
HDL	High-density lipoprotein
HDL-C	High-density lipoprotein cholesterol
HSDA	N-(2-hydroxy-3-sulfopropyl)-3,5-dimethoxyaniline
IQR	Interquartile range
LDL	Low-density lipoprotein
LDL-C	Low-density lipoprotein cholesterol
LDLR	LDL receptor
n	Number
NA	Not applicable
NCHS	National Center for Health Statistics
NHANES	National Health and Nutrition Examination Survey
OR	Odds ratio
PEG	Polyethylene glycol
SD	Standard deviation
TC	Total cholesterol
TG	Triglyceride
VLDL	Very low-density lipoprotein
WHO	World Health Organization
