# Supplementary material for: Role of Circulating Lipids in Mediating the Diabetogenic Effect of Obesity
Source: Biomedicines. 2025 Dec 20;14(1):11. doi: 10.3390/biomedicines14010011 (PMC12837726; doi:10.3390/biomedicines14010011)
Supplement: Supplementary file 1 [file biomedicines-14-00011-s001.zip › biomedicines-4035114-supplementary.pdf]

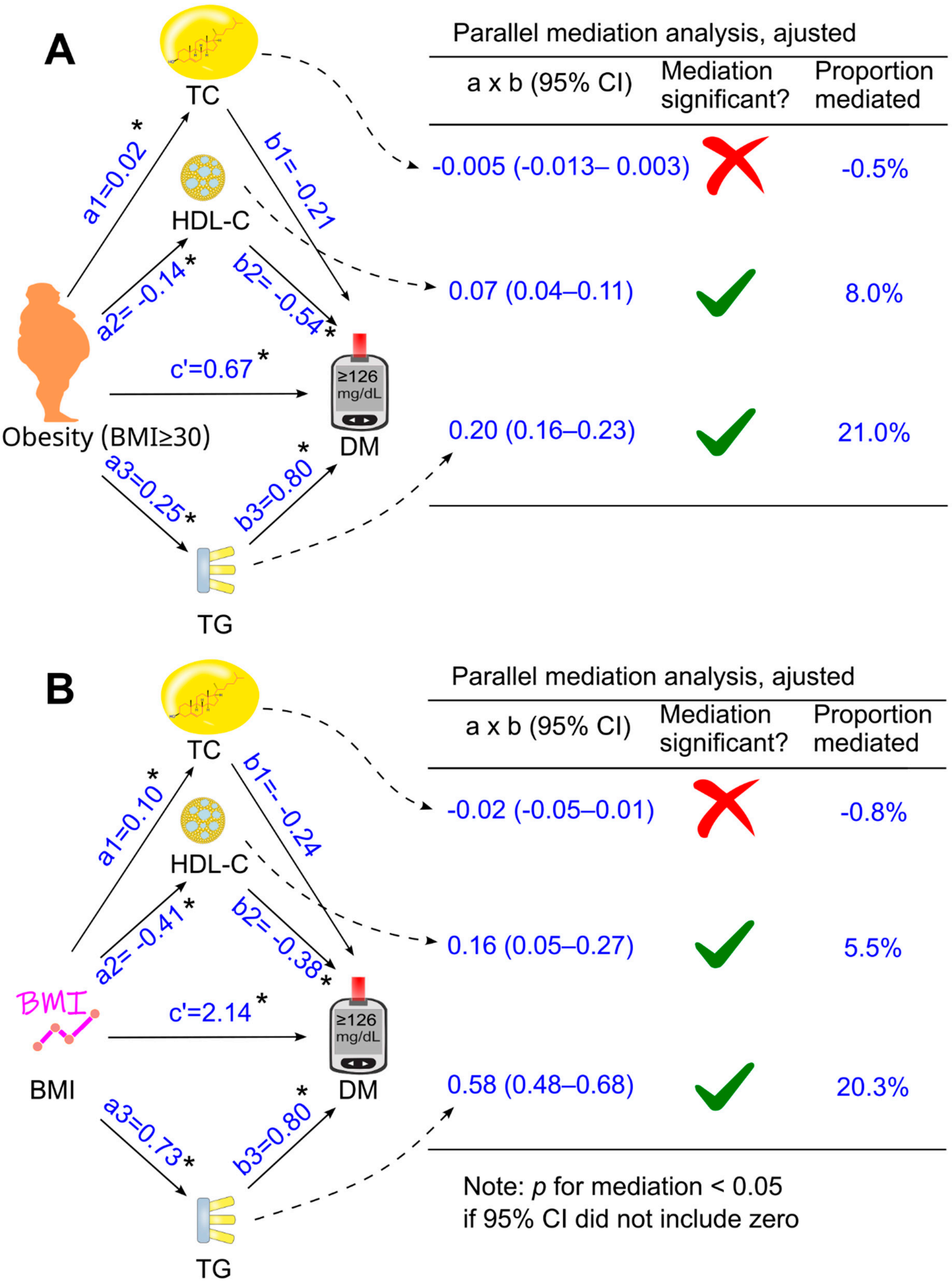

Supplementary Figure S1. Sensitivity analysis of the parallel analysis after excluding participants who were taking anti-diabetic or lipid-lowering drugs. Among all participants, 1939 with known diabetes were excluded as they were receiving anti-diabetic drugs or had an unknown treatment status. An additional 2169 participants with known hyperlipemia were

excluded as they were receiving cholesterol-lowering drugs or had an unknown treatment status. Consequently, the remaining 22,519 participants were included in the sensitivity analysis. Total cholesterol, HDL cholesterol, and triglycerides were placed simultaneously into the analysis as parallel mediators for the effect of obesity or BMI on diabetes. This analysis was adjusted for confounding factors, including age, sex, ethnicity, poverty-income ratio, education, survey period, lifestyle confounding factors (physical activity, alcohol consumption, and smoking status), and clinical confounding factors (hypertension and family history of diabetes). Abbreviations: a, association coefficient between obesity and the tested mediator, or between BMI and the tested mediator; b, association coefficient between the tested mediator and diabetes; c', association coefficient between obesity and diabetes or between BMI and diabetes in the presence of the tested mediators; BMI, body mass index; CI, confidence interval; DM, diabetes; HDL-C, high-density lipoprotein cholesterol; TC, total cholesterol; TG, triglycerides. Green ticks indicate statistical significance, while red crosses indicate non-significance.

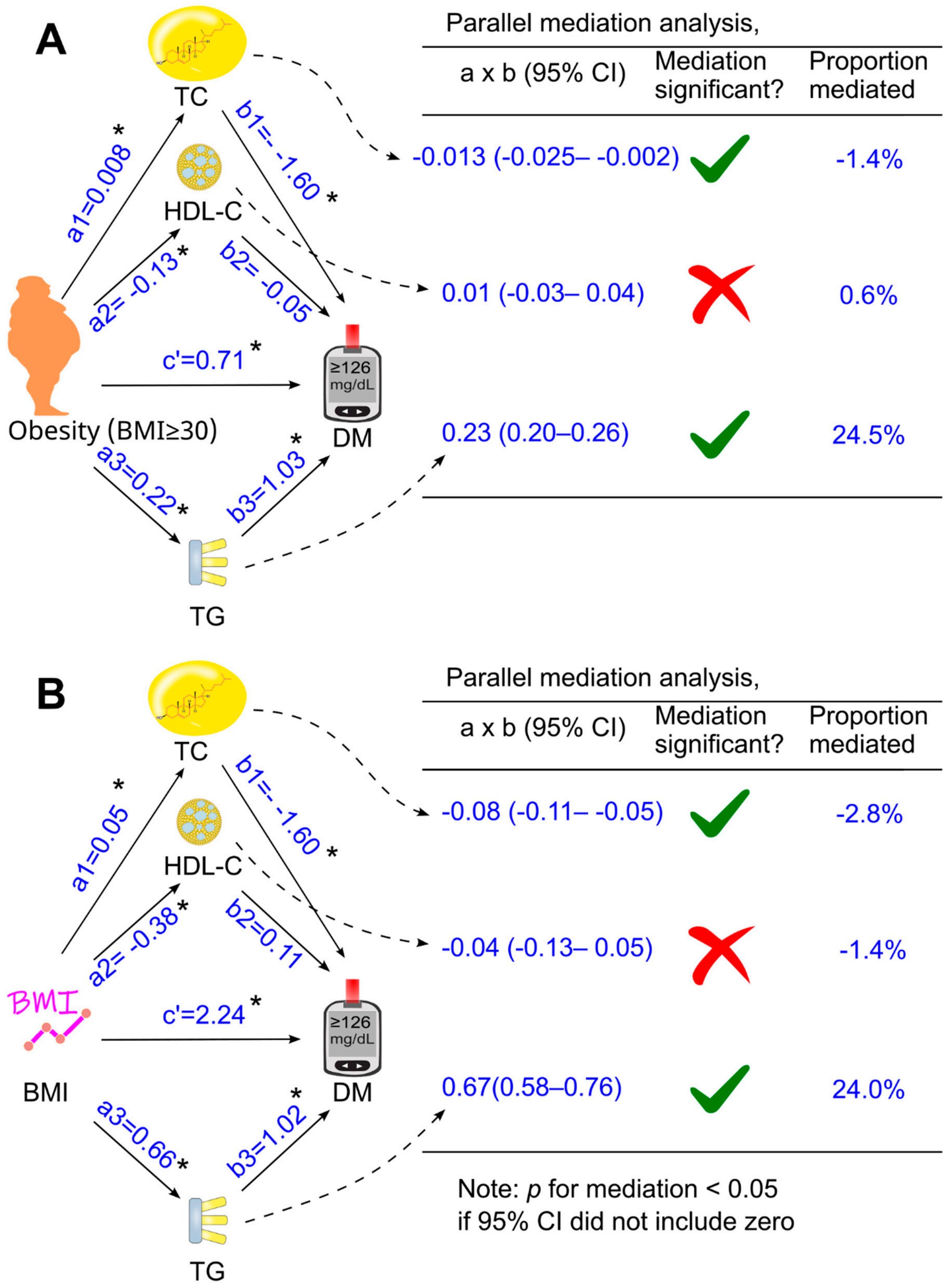

Supplementary Figure S2. Sensitivity analysis of the parallel analysis after excluding participants who participated in surveys prior to 1999. Among all participants, 9428 who participated in surveys prior to 1999 were excluded. Consequently, the remaining 17,199 participants were included in the sensitivity analysis. Total cholesterol, HDL cholesterol, and

triglycerides were placed simultaneously into the analysis as parallel mediators for the effect of obesity or BMI on diabetes. This analysis was adjusted for confounding factors, including age, sex, ethnicity, poverty-income ratio, education, survey period, lifestyle confounding factors (physical activity, alcohol consumption, and smoking status), and clinical confounding factors (hypertension and family history of diabetes). Abbreviations: a, association coefficient between obesity and the tested mediator, or between BMI and the tested mediator; b, association coefficient between the tested mediator and diabetes; c', association coefficient between obesity and diabetes or between BMI and diabetes in the presence of the tested mediators; BMI, body mass index; CI, confidence interval; DM, diabetes; HDL-C, high-density lipoprotein cholesterol; TC, total cholesterol; TG, triglycerides. Green ticks indicate statistical significance, while red crosses indicate non-significance.

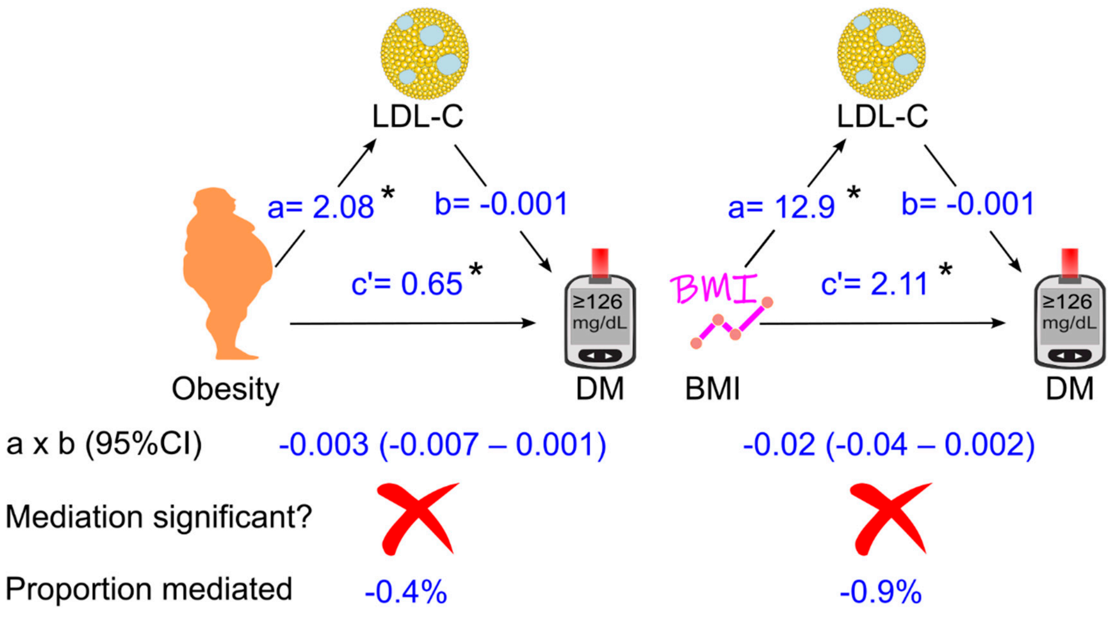

Supplementary Figure S3. Sensitivity analysis of association coefficients of LDL cholesterol for mediating the effect of obesity or BMI on diabetes after excluding those who were taking anti-diabetic or lipid-lowering drugs. Among the LDL cholesterol sub-cohort, 1749 participants with known diabetes were excluded as they were receiving anti-diabetic treatment or had an unknown treatment status. An additional 2076 participants with known hyperlipemia were excluded as they were receiving cholesterol-lowering drugs or had an unknown treatment status. Consequently, the remaining 19,692 participants were included in the sensitivity analysis. The analysis was adjusted for age, sex, ethnicity, poverty-income ratio, education, survey period, lifestyle confounding factors (physical activity, alcohol consumption, and smoking status), clinical confounding factors (hypertension and family history of diabetes), HDL cholesterol, and triglycerides. Abbreviations: a, association coefficient between obesity and LDL cholesterol, or between BMI and LDL cholesterol; b, association coefficient between LDL cholesterol and diabetes; c', association coefficient between obesity and diabetes or between BMI and diabetes in the presence of the tested mediators and confounders. BMI, body mass index; CI, confidence interval; DM, diabetes; HDL, high-density lipoprotein. Red crosses indicate non-significance.

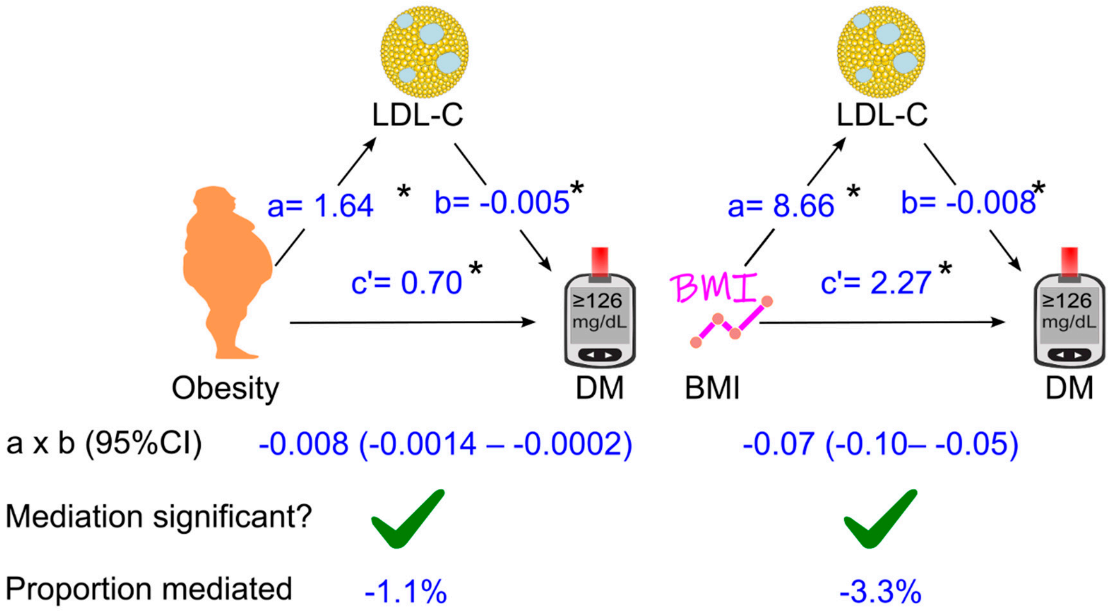

Supplementary Figure S4. Sensitivity analysis of association coefficients of LDL cholesterol for mediating the effect of obesity or BMI on diabetes after excluding those who participated in surveys prior to 1999. Among the LDL cholesterol sub-cohort, 6715 participants who participated in surveys prior to 1999 were excluded. Consequently, the remaining 16,802 participants were included in the sensitivity analysis. The analysis was adjusted for age, sex, ethnicity, poverty-income ratio, education, survey period, lifestyle confounding factors (physical activity, alcohol consumption, and smoking status), clinical confounding factors (hypertension and family history of diabetes), HDL cholesterol, and triglycerides. Abbreviations: a, association coefficient between obesity and LDL cholesterol, or between BMI and LDL cholesterol; b, association coefficient between LDL cholesterol and diabetes; c', association coefficient between obesity and diabetes or between BMI and diabetes in the presence of the tested mediators and confounders. BMI, body mass index; CI, confidence interval; DM, diabetes; HDL, high-density lipoprotein. Green ticks indicate statistical significance.
